# mHealth App Patient Testing and Review of Educational Materials Designed for Self-Management of Gout Patients: Descriptive Qualitative Studies

**DOI:** 10.2196/mhealth.9811

**Published:** 2018-10-15

**Authors:** Amy D Nguyen, Lauren J Frensham, Michael XC Wong, Sylvain MM Meslin, Paige Martin, Annie YS Lau, Melissa T Baysari, Richard O Day

**Affiliations:** 1 St Vincent's Clinical School University of New South Wales Sydney Sydney Australia; 2 Department of Clinical Pharmacology & Toxicology St Vincent’s Hospital Sydney Darlinghurst Australia; 3 Centre for Health Systems and Safety Research Australian Institute of Health Innovation Macquarie University Sydney Australia; 4 Centre for Health Informatics Australian Institute of Health Innovation Macquarie University Sydney Australia; 5 School of Medical Sciences University of New South Wales Sydney Sydney Australia

**Keywords:** mobile apps, gout, self-management, chronic disease, uric acid

## Abstract

**Background:**

Gout is a form of chronic arthritis caused by elevated serum uric acid (SUA) and culminates in painful gout attacks. Although effective uric acid-lowering therapies exist, adherence is low. This is partly due to the lack of support for patients to self-manage their disease. Mobile health apps have been used in the self-management of chronic conditions. However, not all are developed with patients, limiting their effectiveness.

**Objective:**

The objective of our study was to collect feedback from gout patients to design an effective gout self-management app.

**Methods:**

Two descriptive qualitative studies were conducted. In Study 1, researchers developed a short educational video and written materials about gout management, designed to be embedded into an app; 6 interviews and 1 focus group were held with gout patients to gather feedback on these materials. Usability testing in Study 2 involved additional gout patients using a pilot version of *Healthy.me Gout*, a gout self-management app, for 2 weeks. Following the trial, patients participated in an interview about their experiences using the app.

**Results:**

Patients viewed the gout educational material positively, appreciating the combined use of video, text, and images. Patients were receptive to using a mobile app to self-manage their gout. Feedback about Healthy.me Gout was generally positive with patients reporting that the tracking and diary features were most useful. Patients also provided suggestions for improving the app and educational materials.

**Conclusions:**

These studies involved patients in the development of a gout self-management app. Patients provided insight to improve the app’s presentation and usability and general lessons on useful features for chronic disease apps. Gout patients enjoyed tracking their SUA concentrations and gout attack triggers. These capabilities can be translated into self-management apps for chronic diseases that require monitoring of pathological values, medication adherence, or symptoms. Future health app design should integrate patient input and be developed iteratively to address concerns identified by patients.

## Introduction

Mobile health (mHealth) technologies present opportunities for improving patient self-management of chronic diseases [1,2]. Smartphones and tablets are increasingly used worldwide, and more than 100,000 health-related apps are available to users [1]. Studies have shown that user-centered design is key to ensuring that mobile self-management apps are effective in heart health, diabetes, and asthma [3-6]. However, very few apps are codeveloped with end users, limiting their uptake and effectiveness.

Gout is a chronic form of arthritis that causes a significant health burden worldwide [7]. It results from elevated serum uric acid (SUA) concentrations and culminates in debilitating gout attacks [8-10]. Successful gout self-management is dependent on behavioral factors but ultimately upon medication adherence. Highly effective uric acid-lowering therapies (ULTs) are available. However, adherence to ULTs in gout patients is very low; in fact, it has the lowest adherence rate of all chronic diseases [11]. Poor quality educational resources and limited patient knowledge have been identified as key barriers to adequate ULT adherence [12,13]. An intervention that was delivered by nurses comprised personalized education, lifestyle advice, and appropriate prescribing of ULTs resulted in 92% of patients achieving target SUA concentrations 1-year post intervention [14]. Although effective, this intervention was very labor and resource intensive and therefore, unlikely to be sustainable in the long term. A more automatic and far-reaching method of delivering education and lifestyle and treatment advice could be via an app. Previous research has identified that no completely electronic app existed that contained all the required features to support patients in self-managing their gout effectively. One app incorporated these features; however, it required patients to manually complete print-outs to record their SUA and gout attacks [15]. Further, there was no evidence that these apps were developed using patient input. In response to the limitations of the existing gout management apps and the potential for a high-quality app to improve patient self-management of chronic gout, a pilot version of the *Healthy.me Gout* app was developed. *Healthy.me Gout* is an adaptation of *Healthy.me*, a general health app and website, with personal health records, educational information, and Web-based patient support. This study aimed to build an effective gout self-management app by seeking patient input on prototypes of educational material designed to be embedded into the app and to obtain patient feedback of a pilot version of *Healthy.me Gout*.

## Methods

### Ethics

Ethics approvals for these studies were obtained from the University of New South Wales Human Research Ethics Advisory Panel (references #HC16263 and #HC15199).

### Study Design

This paper describes 2 studies that were conducted in parallel. Study 1 explored the opinions of gout patients on the content and format of prototypes of educational material on gout management. In Study 2, gout patients participated in app testing where they were given 2 weeks’ access to an initial iteration of *Healthy.me Gout*, an app designed for patients to self-manage their gout. Following this period, patients participated in an interview about their experiences in using the app.

### Recruitment

To recruit patients with gout, 2 strategies were adopted. The first approach involved contacting general practitioners throughout Sydney, Australia, via email, phone, fax, or in person to distribute invitation letters to their patients with gout. General practitioners were asked to provide the researchers with the contact details of patients who expressed an interest in participating in the study. These patients were then contacted by researchers. This recruitment strategy was supplemented by posting study flyers in pharmacies and medical practices across Sydney and public spaces at St Vincent’s Hospital, Sydney. Rolling recruitment for Study 1 lasted approximately 12 weeks until theme saturation was reached. Recruitment for Study 2 was conducted concurrently. All participants were reimbursed with an Aus $50 gift card. Inclusion criteria for both studies were adults with a diagnosis of gout, and exclusion criteria included cognitive impairment or limited understanding of English such that participation in an interview or focus group would be difficult. Participants in Study 2 also had to have access to a smart device.

### Study 1: Assessing Gout Educational Resources to be Embedded Into a Mobile App

#### Design of Educational Materials

##### Written Information

**Figure 1:**
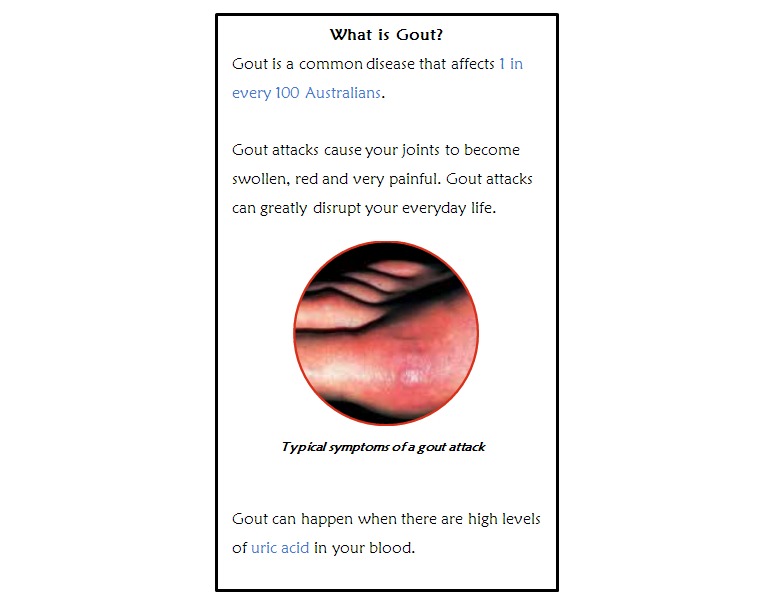
Example of the paper prototype of written educational materials shown to gout patients.

Written educational materials were developed using a combination of resources obtained from consumer group organizations, Australian Rheumatology Association, Arthritis Australia, and the European League Against Rheumatology guidelines for gout management [16]. The materials addressed important aspects of gout and its management. The topics included symptoms of gout attacks, diagnosis of gout, hyperuricemia as a cause of gout, risk factors for gout, consequences of chronic hyperuricemia, acute gout treatments, use of ULTs and the importance of ULT adherence, and prophylaxis during ULT initiation. The research team, which included a senior rheumatologist, reviewed the information to validate the content. The information, written in plain English, was embedded into paper prototypes to simulate how the information would appear on a smart device screen in terms of the size and location of text, as seen in Figure 1. Blue-colored text was used to indicate hyperlinked keywords, simulating how users would be able to use these to click to different pages in the app. Images were included in the written materials.

##### Animated Video

**Figure 2:**
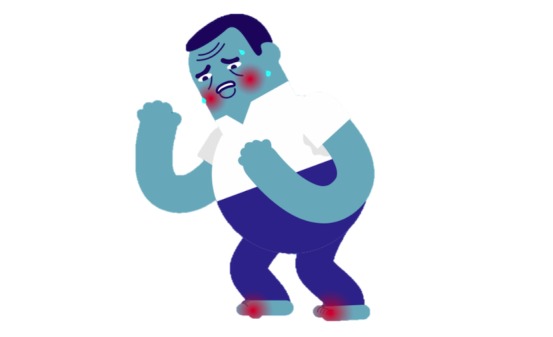
"Gout Man" as depicted in the animation video.

A 2-minute animated video was developed to introduce gout management and highlight the importance of adherence to ULTs. The video was designed to be conversational in tone and employed a persona, “Gout Man,” who depicted a stereotypical gout patient, as seen in Figure 2.

**Figure 3:**
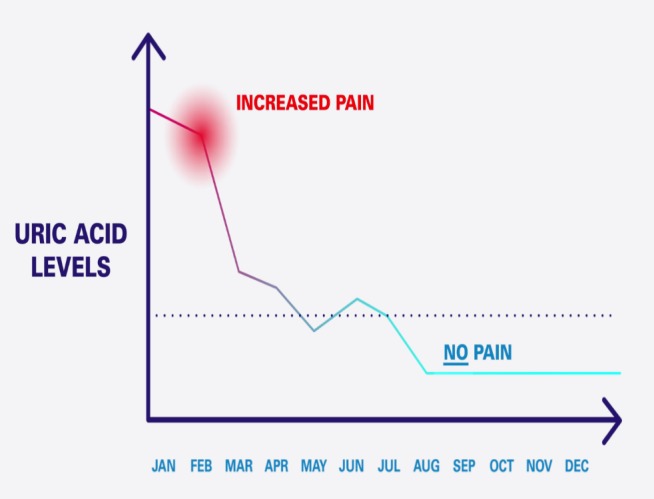
Graph of serum uric acid tracker shown in the educational animation video.

The video included a graph that represented monitoring SUA over time, as seen in Figure 3. The graph highlighted the relationship between SUA and gout attacks, demonstrating that as SUA concentrations decrease, the likelihood of pain from gout attacks decreases. The target for SUA was displayed on this graph as a perforated line.

#### Interviews and Focus Groups

Patients were provided with the option of attending either an interview or focus group based on their preference and convenience. Using a semistructured interview guide (Multimedia Appendix 1), 6 one-on-one interviews (average duration, 45 minutes; range, 15-56 minutes) and 1 focus group (with 5 patients, 102 minutes in duration) were held to obtain feedback on the educational materials. Broadly, patients were asked questions covering their opinions of the presentation and content of the written and video educational resources. All interviews were carried out by a fourth-year medical student, and the focus group was cofacilitated by a PhD research student.

#### Analysis

The interviews and focus group were audiotaped and transcribed verbatim for analysis. Thematic analyses of the transcripts were carried out concurrently but independently by 2 researchers (AN and MW) to determine when thematic saturation occurred [17]. Transcripts from approximately half of the participants were initially reviewed and coded for themes after which researchers convened and developed a coding framework using an inductive approach, allowing for the most predominant themes to be identified. Emerging themes were categorized into patient perspectives of the educational materials and suggestions for improvements. All transcripts were then reanalyzed using this framework and coded under the predetermined themes; 2 coders resolved any discrepancies by consensus.

### Study 2: Pilot Testing of the Gout Self-management Mobile App

#### Healthy.me Gout Design

**Figure 4:**
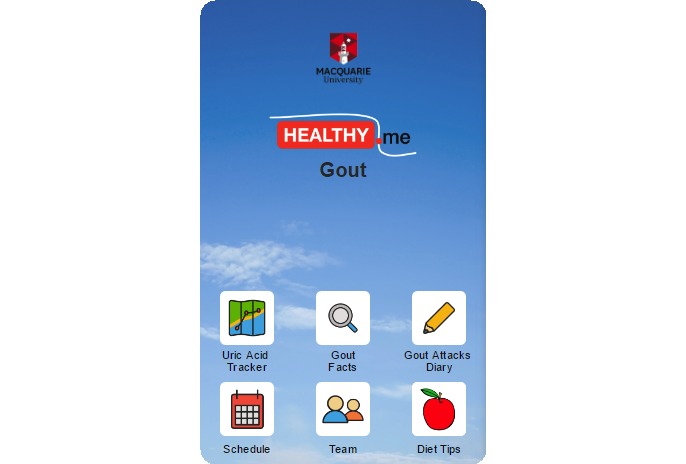
Screenshot of *Healthy.me Gout* home screen. Source: Image created by the authors, together with “The Explainers”.

The written materials and animated video were incorporated into an app called *Healthy.me Gout*. *Healthy.me Gout* is a modified version of *Healthy.me*, a general health app that has been shown to improve influenza vaccination rates and address the needs of breast cancer survivors [18-20]. *Healthy.me Gout* was developed after interviews with patients regarding their current gout management [21], a review of commercially available apps for patient self-management of gout [15], and subsequent feedback from gout patients on these apps and the generic version of *Healthy.me* [22]. *Healthy.me Gout* contained 6 features, as depicted in Figure 4 and summarized in Table 1.

#### Two-Week Use of App

Patients were asked to use *Healthy.me Gout* for 2 weeks and explore each feature of the app to assess for app usability. Patients were advised that they would be emailed 4 mock SUA values approximately 4 days apart for them to enter into the “Uric Acid Tracker” feature of the app, and these SUA values were 0.70, 0.50, 0.34, and 0.25 mmol/l, respectively. Because it was unlikely that patients would have their SUA measured during the 2-week trial period, this allowed patients to experience inputting data and visualizing SUA values being plotted on the “Uric Acid Tracker” graph. Throughout the 2-week period, activity log data were collected in the background with times of patient log-in and use of the features recorded.

**Table 1:** Features of *Healthy.me Gout.*

Feature	Summary
Uric Acid Tracker	Patient’s serum uric acid concentrations represented in a graph over time and in relationship to the target serum uric acid concentration (0.36 mmol/l)
Gout Facts	Educational materials including brief written information with hyperlinks and images, and animation video about gout and its management
Gout Attacks Diary	Ability to record gout attacks including pain intensity, the location of attack(s), and trigger(s)
Schedule	Option for patients to set reminders such as to take their medications, have blood tests, record gout attacks, and see their general practitioner
Team	Option for patients to input contact details (eg, of their general practitioner, pathology service, or researchers)
Diet Tips	Evidence-based list of foods that raise and lower serum uric acid

#### Interviews

Following the 2-week pilot, patients participated in a short interview conducted by a postdoctoral researcher (average duration, 26 minutes; range, 15-33 minutes). The interview guide is presented in Multimedia Appendix 2. Patients were asked about their experiences of using *Healthy.me Gout*, including any challenges faced and the perceived benefits of the app.

#### Analyses

The interviews were audiotaped and transcribed verbatim. The review and coding of themes were carried out by 2 postdoctoral researchers (AN and LF). The researchers discussed the themes that were identified individually and agreed upon the main themes that arose. Activity log data were tabulated to calculate patient usage of the app (ie, the number of times each feature of the app was accessed and by which patient).

## Results

### Study 1: Assessing Gout Educational Resources to be Embedded Into a Mobile App

Overall, 11 gout patients participated in Study 1, at which point theme saturation was reached; 5 patients were recruited via their general practitioners, and 6 responded to flyers. All 11 patients were male and aged between 32 and 85 years (mean, 60 years). The time since their gout diagnosis ranged from 3 to 25 years with 8 patients reporting having experienced 2 or more attacks of gout in the past year. Although all 11 patients owned a computer with access to the internet and were frequent users, only 8 reported owning a smartphone. Patients provided their opinions on the content and presentation of the information shown in the video and written formats (Textbox 1). Patients also provided suggestions for improvement to the written materials and educational video, summarized in Textbox 2.

### Study 2: Pilot Testing of the Gout Self-Management Mobile App

Overall, 5 patients were provided access to *Healthy.me Gout*. Of these 5, 3 also participated in Study 1. The patients were all male and were between the ages of 50 and 69, except for 1 who was younger than 40 years old. The patients had had gout for an average of 7 years and reported experiencing 4 attacks, on average, in the last year. All gout patients in Study 2 owned a smartphone and computer and accessed the internet frequently. Patients were interviewed following their use of *Healthy.me Gout*, and the findings are summarized in Textbox 3.

**Patient feedback on gout educational resources; example quotes are in italics.**

**Presentation**

1. Liked the combination of videos, images, and written text
2. Found informal and conversational tone of video comforting and fostered a sense of inclusion
  - *It makes you feel like you’re not completely out on your own.* [P8]
3. Amount of text in the written material was appropriate
  - *You don’t lose interest.* [P4]
  - *Provided you get the message across it’s fine. It’s not overkill.* [P4]
4. Appreciated video’s length, stating it was short and succinct
5. Concerned about the font of written material being too small, especially when displayed on a mobile phone screen

**Content**

1. The written material described gout well and focused on the importance of regularly taking uric acid-lowering therapies
  - *Gout is curable; you just need to take your pill.* [P8]
2. Images were accurate (ie, similar to patient experiences with gout attacks)
3. List of foods that could trigger gout attacks was not useful, as food triggers are inconsistent across gout patients, and patients were already aware of their dietary triggers
  - *Yeah, that’s [other patient’s trigger] different with me. They tell me you shouldn’t eat oysters and mussels, but I find when I’m eating them I don’t have the attacks.* [P9]
4. Liked tips in written material for remembering to take medications
  - That’s not a bad idea, putting it [medications] next to the toothpaste. [P7]
5. Including the target serum uric acid concentration motivated patients to adhere to medications, and patients liked it visually represented as a line on the graph in the video
6. “Gout Man” seen as representative of gout patients in the community, and having a persona helped patients relate to video’s message
  - *Yeah, he [Gout Man] looks like me.* [P11]

**Suggestions from patients to improve presented gout educational resources; example quotes are in italics.**

**Presentation**

1. Include dropdown menus to hide or expand written material, enabling personalization of information
  - *Your choice, you’re in charge of how much you read.* [P4]
2. Search bar to navigate through written material more efficiently

**Content**

1. More emphasis on permanent joint damage resulting from long-term untreated gout in video and written material
  - *I’ve seen some terrible joints affected by gout that’s been left untreated, and I thought if I don’t want to end up in that situation, I’d go on permanent medication.* [P4]
2. Include effects of gout on daily life in the written material
  - *Trying to walk, it was difficult. So, yeah, how it affected me is what sticks in my mind.* [P1]
3. Greater number of more alarming and graphic images in the written material
  - *You need more horrific pictures, that’s for sure.* [P7]
  - *The only way you’re going to really impact and scare people is with pictures of tophi deposits.* [P3]
  - *What about a picture of that white blood cell skewered by a little crystal? It’ll explain why it’s just like having a fork driven into it.* [P3]

**Summary of patient perceptions and suggestions for improvement of *Healthy.me Gout*.**

**Patient perceptions**

- Straightforward and easy to use
- Appreciated being able to monitor their gout with “Uric Acid Tracker”
- Visualization and feedback of being in the “Danger Zone” of serum uric acid (SUA) concentrations was useful
- “Gout Attacks Diary” useful to see patterns and trends of attacks
- “Team” useful only for contact details of nonregular healthcare providers (eg, specialists)

**Patient suggestions for improvement**

- Automatic input of SUA values into “Uric Acid Tracker”
- “Gout Attacks Diary” determines trends in attack triggers and generates summaries of attacks
- “Schedule” reminders pop up on mobile phone screens, rather than sent via email, and are integrated into device’s native calendar
- Ability to personalize the list of foods to avoid in “Diet Tips”

#### Patient Perceptions of Healthy.me Gout

Patients perceived the app to be straightforward and easy to use. Patients appreciated the ability to monitor their condition using the “Uric Acid Tracker” available within the *Healthy.me Gout* app. Being able to determine whether they were in the “Danger Zone” of SUA concentrations was liked, as was the graph providing individualized feedback with prompts to see their doctor if they were out of range. Similarly, patients found the “Gout Attacks Diary” useful to record their gout attacks, allowing patients to see patterns and trends in their gout attack triggers and to report attacks to their doctors at a later date. One patient explained,

With any medical condition...if one is relying on memory...It’s hard to remember. Whereas if the gout app retains those records then it’s a great help to look at the current condition.

P17

The sliding scale to record pain intensity was also viewed positively. Similar to patients in Study 1, those in Study 2 appreciated that “Gout Facts” was written in simple language with sentences of appropriate length that were easy for them to understand; one patient described,

It’s [the written information] good because they’re apt sentences but they’re not lengthy ones, and in reading an app or data...it’s a real turn-off to read an inch or a long, long paragraph — so, short and sweet sentences are great.

P17

Patients reported that the content included in the “Gout Facts” and “Diet Tips” sections was useful and that the still images served to help the patients visualize what was described in the text. In contrast, the animated video incorporated into “Gout Facts” was described as too general and would only be viewed once.

The “Team” feature of the app was noted to be useful for the contact details of nonregular healthcare providers, such as specialists. However, patients did not find this feature useful otherwise, particularly for including contact details of their general practitioners who they regularly visit.

#### Patients’ Suggestions for Improvements to Healthy.me Gout

Gout patients expressed a preference for their SUA values being automatically updated in the “Uric Acid Tracker” because they usually do not have access to this result unless they are with their doctor. A patient explained,

The results don’t come to me so I won’t actually know what my uric acid level is until I’m sitting opposite Dr [name withheld]...I don’t get a copy of the report. So, yeah, automatically inputted in.

P16

For the “Gout Attacks Diary,” gout patients suggested that this record-keeping feature would be more useful if it could summarize patterns in the triggers of their gout attacks. This information would be helpful in identifying foods or situations for the patient to avoid. One patient elaborated,

If you have an attack it’s probably good to see if there’s a pattern...if there’s certain times of the year or certain triggers. It would be nice to use it in the long term.

P13

Patients suggested that if a report of their gout attack history were generated, it would be useful to take to their doctor with a patient describing,

If the individual entries can generate a report, that is either printable or emailable to the doctor; that makes sense to me. In other words, it’s creating a history.

P16

“Schedule” and “Team” were the features least liked by patients. Regarding the “Schedule” feature, patients did not like that the reminders arrived as emails and would have preferred it if they popped up on their mobile phone screens. In particular, reminders to take ULTs on a daily basis were seen as potentially useful for patients with medication adherence issues. Incorporation of these reminders into the device’s native calendar was suggested as a way to integrate the “Schedule” feature into patients’ current use of their smart devices. As smart devices have contact lists, patients reported that the “Team” feature was redundant. A patient reported,

To be honest, I don’t really see the purpose of this one ‘cause if I am going to call my [general practitioner], I can just go to my phone book and just go to dial the [general practitioner].

P14

“Diet Tips,” which was a list of foods that they could or should not eat, was perceived as a useful feature, but patients wanted to personalize the list. This was because the food triggers of gout attacks are highly individual. One patient expressed,

I really would like to have [a list that] is relevant to me, so...proven high purine fruit and veg and drinks, yes, but then allow me to create my personal, relevant lists...so as I’m putting in an attack I can access the relevant triggers that have caused me issues in the past.

P16

Some patients also stated that the list of restricted foods was not extensive enough.

#### Continuity of Use

**Figure 5:**
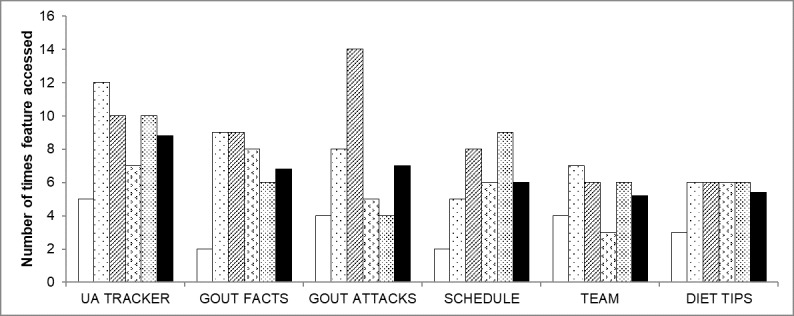
Number of times each feature of Healthy.me Gout was accessed by patients during the 2-week pilot. UA: uric acid.

Patients reported that they would continue to use the app with access to the “Uric Acid Tracker” and “Gout Attacks Diary” being stated as 2 of the main drivers for this. App usage data showed that the “Uric Acid Tracker” was the most accessed feature of the *Healthy.me Gout* app, as seen in Figure 5, and that, on average (final bar of each feature), patients (individually represented as the first 5 bars of each app feature) accessed the app on 5 separate days in the 2-week trial period (range, 4-7; data not shown).

Patients said that they would recommend the app to other gout patients, particularly newly diagnosed gout patients, those who were interested in tracking their SUA, and those who wanted to identify their gout attack triggers. A patient stated,

If you’ve just been diagnosed, it’s got some good facts, and if you’re interested in working out some patterns, it’s good.

P13

Another patient agreed saying,

I think newly diagnosed people can learn more from the gout facts, and also tracking their uric acid index...this is pretty straightforward, answers all your questions about gout, so I think it will be good.

P14

## Discussion

### Principal Findings

This study demonstrated the importance of involving patients in the design and development of a self-management app for gout patients. Patients said that they were open to using an app to support the self-management of their condition. Patients reported that the most useful features of the app were the ability to monitor their SUA, record gout attacks, and access educational resources. Importantly, patients provided feedback to improve the app and the educational material embedded within it.

Previous studies have shown that incorporating images into educational materials increases their effectiveness specifically by improving patient attention, comprehension, recall, and adherence to the content of the materials [23]. Employing a health app to deliver patient education, as opposed to paper-based formats (eg, leaflets and booklets), enables the use of a combination of media to engage patients in learning about their disease, which was noted as important by patients in this study with patients being particularly receptive to the dynamic educational video.

A self-management app that facilitates longer-term management is important for chronic diseases. Patients expressed that the “Uric Acid Tracker” and “Gout Attacks Diary” in *Healthy.me Gout* were valuable tools to enable them to track their disease over time. However, enhancing the tool by incorporating features, such as automatic input of SUA values, ideally through data linkage with electronic patient records, may encourage patients to continue using the app long term. The integration of *Healthy.me Gout* with native features of smart devices, such as the calendar and contacts list as well as enabling in-device pop-up alerts, were seen to be important for incorporating the app into the patients’ daily lives, which could improve the longevity of app use. Indeed, the “Team” and “Schedule” features of the app were not well liked because they did not integrate with native apps of devices.

Successful behavior change using self-management strategies should include not only education but also support through tools, such as alerts and personalized content and feedback [24,25]. This was reflected in our study with the ability for patients to personalize their *Healthy.me Gout* app viewed as being important; for example, patients wanted to create personalized lists of potential food triggers in “Diet Tips” and liked that they could collapse (if irrelevant) or expand (if relevant) information boxes in the written educational materials. In this way, patients could customize their learning journey. Similarly, patients in this study wanted the app to produce personalized summaries of their clinical gout symptoms to share with their healthcare providers during medical consultations. The findings of this study are similar to those of other mHealth app usability studies in which patients with other chronic conditions have stressed the importance of health apps being able to support patient-doctor communication during clinical encounters [26].

Involving end users in the development of apps can improve effectiveness [3-6,26]. However, not all app developments incorporate this. By incorporating the feedback from gout patients in this study to modify the *Healthy.me Gout* app, it is anticipated that use of the app will be substantially improved, subsequently increasing app uptake and improving long-term gout management. Because most health app evaluations are predominantly usability studies, the ability of an improved iteration of *Healthy.me Gout* to support patient self-management of gout will be evaluated in a larger randomized controlled trial, wherein gout patients will use the app for a longer period and clinical outcomes will be assessed [27,28]. This is of particular importance given the movement toward the promotion of greater use of digital health interventions, particularly with healthcare providers now being encouraged to integrate patient-focused mHealth apps into their delivery of patient care [26,29].

### Limitations

Given that gout is a chronic condition and the studied patients presented with varying degrees of gout severity, being restricted to a 2-week period of app use meant that it was unlikely that all patients would have a gout attack or have SUA measured in this time. Thus, interactions of patients with the features of the app were highly variable. Further studies with longer monitoring of app usage are required to identify features that are utilized by gout patients in the longer term. In this study, the app was tested by only 5 gout patients who were all male, owned a smartphone, and accessed the internet frequently. Because differences among treatment regimens, treatment monitoring, and existing comorbidities are present between male and female gout patients, further app testing in female gout patients is required [30]. Testing the app with patients with varying characteristics would enable the provision of further evidence of the feasibility of the app for a broader gout population. Additionally, the participants were a self-selecting sample. Patient responses to the recruitment strategies likely reflect higher levels of interest in learning more about their gout diagnosis or using self-management health apps and, for Study 2, required patients to have access to a smart device to participate.

### Conclusions

Experiences from this study provide important insights for the development of future self-management mHealth apps. This paper demonstrates the importance of integrating patient input into health app design and iterative development to identify and address the concerns of affected patients. Similar to many other chronic conditions, optimal management of gout requires the monitoring of a pathological value or surrogate indicator of risk, namely, SUA. SUA is influenced by medication adherence and monitoring its concentration reduces the risk of gout attacks from occurring by promoting adherence. The use of graphs to display the pathological value and risk of symptoms that occur as well as a facility to record symptoms, as in this gout app, were highly valued by patients. Future self-management apps for other chronic conditions should involve patients in their design and development and also incorporate similar features to present the relationship between a pathological value and risk of a negative clinical outcome. Additionally, once an app is deemed user friendly by end users, it is necessary to undertake clinical evaluations to determine the health benefits of introducing apps into routine healthcare.

## Appendix

Multimedia Appendix 1 Interview guide to obtain feedback on developed prototypes of gout educational materials.

Multimedia Appendix 2 Interview guide for post-two-week use of Healthy.me Gout.

## Abbreviations

SUA: serum uric acid
ULT: uric acid-lowering therapy
