# Supplementary material for: mHealth App Patient Testing and Review of Educational Materials Designed for Self-Management of Gout Patients: Descriptive Qualitative Studies
Source: JMIR Mhealth Uhealth. 2018 Oct 15;6(10):e182. doi: 10.2196/mhealth.9811 (PMC6305897; doi:10.2196/mhealth.9811)
Supplement: Multimedia Appendix 1 [file mhealth_v6i10e182_app1.pdf]

Appendix A – Interview guide to obtain feedback on developed prototypes of gout educational materials

1. What format would you like the information in the app to be presented (e.g. text, videos etc.)? Why?

We are in the early stages of developing a short video on gout for our app.

2. How would you like gout information to be displayed to you in a video (e.g. real people speaking or an animation or just text)? Why?
3. Now that you have watched the video, what do you feel is the main message of the video?
4. What do you like about the video? What aspects could be improved?

We have also developed some written material that will accompany the video. Here are some examples of the layouts we will be using.

5. What do you like about the written materials? What could be improved?
6. Between the video and written materials, which format do you prefer? What qualities make it better than the other one? Would you like a mix of the formats?

Are there any topics of concern that you feel are not adequately addressed in either of the resources? Can you elaborate?
