# Supplementary material for: mHealth App Patient Testing and Review of Educational Materials Designed for Self-Management of Gout Patients: Descriptive Qualitative Studies
Source: JMIR Mhealth Uhealth. 2018 Oct 15;6(10):e182. doi: 10.2196/mhealth.9811 (PMC6305897; doi:10.2196/mhealth.9811)
Supplement: Multimedia Appendix 2 [file mhealth_v6i10e182_app2.pdf]

Appendix B – Interview guide for post-two-week use of *Healthy.me Gout*

1. Tell me about your experiences using the Healthy.me Gout app.
2. Describe your experience with learning how to use the app.
3. Specific features – what is your experience with specific app features:
   a. Uric acid tracker
   b. Gout attack diary
   c. Written information on gout
   d. Video animation
   e. Reminders
   f. Team
4. What would you ask the developers to change? e.g. colours, layout, icons, font, font size
5. Would you continue to use the Healthy.me Gout app if it were available? What would you use it for?
6. Would you recommend it to other gout patients? If so, which ones?

Is there anything that we haven't talked about that you think is important to share?
